# The direct and indirect effects of length of hospital stay on the costs of inpatients with stroke in Ningxia, China, between 2015 and 2020: A retrospective study using quantile regression and structural equation models

**DOI:** 10.3389/fpubh.2022.881273

**Published:** 2022-08-12

**Authors:** Ming Su, Dongfeng Pan, Yuan Zhao, Chen Chen, Xingtian Wang, Wenwen Lu, Hua Meng, Xinya Su, Peifeng Liang

**Affiliations:** ^1^School of Public Health, Ningxia Medical University, Yinchuan, China; ^2^Department of Emergency Medicine, People's Hospital of Ningxia Hui Autonomous Region, Yinchuan, China; ^3^Department of Medical Records and Statistics, People's Hospital of Ningxia Hui Autonomous Region, Yinchuan, China

**Keywords:** costs, length of hospital stay (LOS), stroke, quantile regression, structural equation models (SEMs)

## Abstract

**Importance:**

Length of hospital stay (LOHS) is the main cost-determining factor of hospitalization for stroke patients. However, previous analyses involving LOHS did not consider confounding or indirect factors, or the effects of other factors on LOHS and inpatient costs.

**Objective:**

To investigate the direct and indirect effects of LOHS on the hospitalization costs of inpatients with ischemic and hemorrhagic stroke.

**Design, setting, and participants:**

This was a population-based, retrospective, and observational study that analyzed data acquired from the Nationwide Inpatient Sample between 2015 and 2020 relating to ischemic and hemorrhagic stroke in Ningxia, China.

**Main outcomes and measures:**

Hospitalizations were identified by the International Classification of Diseases 10th Revision (ICD-10). Inpatient costs were described by the median M (P25, P75). We used a quantile regression model to estimate the linear relationships between a group of independent variables X and the quantile of the explained variable hospitalization cost (Y). A structural equation model (SEM) was then used to investigate the direct and indirect effects of LOHS on inpatient costs.

**Results:**

The study included 129,444 patients with ischemic stroke and 15,525 patients with hemorrhagic stroke. The median LOHS was 10 (8–13) days for ischemic stroke and 15 (10–22) days for hemorrhagic stroke. The median M (P_25_, P_75_) of inpatient costs was $1020 (742–1545) for ischemic stroke and 2813 (1576–6191) for hemorrhagic stroke. The total effect of LOHS on inpatient costs was 0.795 in patients with ischemic stroke. The effect of yearof discharge (X4) and CCI (X8) on inpatient costs was dominated by an indirect effect through the LOHS. The indirect effect was −0.071 (84.52% of the total effect value) and 0.034 (69.39% of the total effect value), respectively. The total effect of LOHS on inpatient costs in patients with hemorrhagic stroke was 0.754. The influence of CCI on inpatient costs was dominated by an indirect effect through LOHS; the indirect effect value was −0.028 (77.78% of the total effect value). The payment type, surgery, method of discharge, and hospital level also exerted an impact on inpatient costs by direct and indirect effects through the LOHS.

**Conclusions and relevance:**

Length of hospital stay (LOHS) was identified as the main factor influencing hospitalization costs. However, other social factors were shown to indirectly influence hospitalization costs through the LOHS. Taking effective measures to further reduce hospitalization costs remains an effective way to control hospitalization costs for stroke patients.

## Question

In addition to the direct effect on hospitalization costs, do other social factors indirectly affect hospitalization costs through the length of hospital stay (LOHS)?

## Findings

This was a retrospective study of hospitalizations involving 129,444 cases of ischemic stroke and 15,525 cases of hemorrhagic stroke. We found that LOHS was the main factor that influenced hospitalization costs. Other social factors also had an impact on inpatient costs *via* direct and indirect effects acting on the LOHS, including year of discharge, Charlson Comorbidity Index (CCI), payment type, surgery, discharge method, and hospital level.

## Meaning

Length of hospital stay (LOHS) not only exerts direct effects on the cost of hospitalization; it also exerts indirect effects on other social factors, thus suggesting that taking effective measures to further reduce hospitalization costs remains an effective way to control hospitalization costs for stroke patients.

## Introduction

The Global Burden of Disease study in 2016 estimated that the overall lifetime risk of stroke in the Chinese population was 39.9%, thus ranking first in the world (1). The combination of aging in the population of China and the high prevalence of risk factors has created a significant trend for stroke in China (2–4). The standardized rate of the first stroke among Chinese residents aged 40–74 years has increased by a mean of 8.3% per year (5). Furthermore, it is estimated that among the residents of China who are aged 40 years, there are 13.18 million stroke patients and more than 1.9 million residents die of stroke annually (5). Although China changed its one-child policy in 2016, the aging trend is unlikely to be reversed in the short term. This trend will place significant pressure on the public health system.

Over the past 30 years, China's economy has achieved unprecedented growth; medical health provision and public health expenditures have also grown rapidly. The observed growth in medical expenses has exceeded that of economic growth. It is also evident that the out-of-pocket medical expenses of patients are gradually decreasing. Since 2007, China has implemented comprehensive medical reforms with medical insurance coverage increasing from 45% in 2006 to more than 95% in 2017 (6, 7). Expanding the coverage of medical insurance can increase the detection rate of strokes and the chance of patients receiving treatment, thus increasing the expenses associated with diagnosis and treatment. The Report on Cardiovascular Health and Diseases in China 2020 showed that the total hospitalization expenses for ischemic stroke were $10.469 billion while that for intracranial hemorrhage was $43.315 billion in 2018 (8). Excluding the impact of price factors, the mean annual growth rate of hospitalization expenses for ischemic stroke and intracranial hemorrhage since 2014 were 18.65 and 14.00%, respectively (8).

Previous research has provided strong evidence that length of hospital stay (LOHS) is the main cost-determining factor for hospitalization in stroke patients (9–14). However, the association between LOHS and inpatient costs has been largely ignored. For example, comorbidities can both prolong the LOHS and increase the cost of hospitalization. Furthermore, LOHS exhibits a close relationship with both hospitalization and inpatient costs, thus implying that comorbidities can affect inpatient costs *via* both factors. However, most researchers still use traditional linear regression models to analyze the influence exerted by inpatient costs (12, 15–17); these models can only explain the direct effect between independent and dependent variables. Consequently, it is very difficult to select an appropriate set of variables by considering a single regression model. However, traditional linear regression models are associated with limitations with regard to their ability to analyze data arising from the skewed distribution of inpatient costs. These models also ignore the effect of the distribution status of covariates on the dependent variable. In this study, we aimed to investigate the relationship between different clinical characteristics and LOHS, inpatient costs, and the relationship between LOHS and inpatient costs. In other words, we investigated the direct and indirect effects of LOHS on inpatient costs in order to provide further options to reduce the economic burden of patients with stroke.

## Methods

### The registration and approval of standard protocols

We designed a population-based and retrospective observational study that utilized data from the Nationwide Inpatient Sample (NIS). The medical records manager concealed the names, ID numbers, phone numbers, and addresses of inpatients before we extracted the data; each patient was identified by a unique medical records number. The ethics review committee of The People's Hospital of Ningxia Hui Autonomous Region approved this study (Approval number: 2020-KY-053).

### Data abstract

We extracted the first page of each inpatient medical record from 2015 to 2020 in Ningxia, located in the northwest of China; the medical resources and comprehensive medical quality of this hospital were ranked 14^th^ in China's 31 provinces. We collected all discharged patient variables from the first page of medical records in Grade 2 medical and health institutions (providing comprehensive medical and health services and undertaking certain teaching and research tasks of regional hospitals with 101–500 beds) and Grade 3 medical and health institutions (providing high level specialized medical and health services to several regions along with higher education and research tasks in regional hospitals with >500 beds). The first pages of the inpatient medical record, including medical record number, demographic characteristics, primary, primary and secondary diagnoses, procedures, length of stay (LOHS), and method of payment were collated; in total, 235 variables were acquired.

According to the International Classification of Diseases 10th Revision (ICD-10), patients with a primary diagnosis of hemorrhagic stroke (coded as I60-I62) and ischemic stroke (coded as I63) were selected. In order to avoid the impact of extreme values in inpatient costs, we excluded 808 observations (accounted for 0.41% of the total number of observations) with LOHS < 1 day or ≥ 180 days, 897 observations (accounted for 0.46%) with inpatient costs < 100 Renminbi (RMB) or > 300,000 RMB and 1,628 observations (accounted for 0.84%) aged < 20 years or > 90 years. In addition, we excluded 20,417 observations (accounted for 10.58%) that were lacking important data such as age, sex, discharge mode, and method of payment.

### Assessment of comorbidity

Stroke patients often experience complications that are defined as a clinical condition that exists at the time of the onset event that is likely to influence inpatient costs. In the present study, we used the Charlson Comorbidity Index (CCI) as a measure to evaluate the severity of comorbidity in stroke patients (Appendix 1).

### Statistical analyses

In order to eliminate the interference of price factors on the inpatient costs of stroke patients in different years, before performing hospitalization cost analysis, we discounted the hospitalization cost by the Consumer Price Index (CPI), taking 2020 as the benchmark year. As inpatient costs followed a skewed distribution, we described these data as a median M (P25, P75). Usually, the influence of X on Y (Y is quantitative) uses linear ordinary least squares (OLS) regression to determine the significance of the regression coefficient and the influence of the direction. However, OLS regression is unable to determine this relationship. OLS regression requires the dependent variable Y to be normally distributed. OLS regression is also sensitive to outliers and problems related to a heterogenous variance. In comparison with OLS regression, quantile regression can investigate the influence of X on Y and provide strong robustness to outliers, dependent variable normality, or heteroscedastic problems.

The quantile regression model is a modeling method that is used to estimate the linear relationship between a group of regressors X and the quantile of the explained variable Y, thus emphasizing changes in the conditional quantiles (18, 19). Quantile regression is simply stated as follows: a continuous random variable y, the overall t quantile is y (t) is defined as: the probability of y y (t) is t, namely t = P [y y (t)] = *F*_[y(t)]_ where P indicates the probability, and *F*_[y(t)]_ indicates the cumulative (probability) distribution function of y. In this study, we used the web version of the Data Science Algorithm platform tool SPSSAU (https://spssau.com/en/index.html) for quantile regression. The inpatient costs were taken logarithmically and then included in the quantile regression model as a dependent variable for analysis. Payment mode, age, patterns of admission, year of admission, length of stay, CCI, surgery, pattern of discharge, and hospital level were set as the independent variable X; the quantile point of the explained variable hospitalization cost (Y) was divided into 10 sections, ranging from 0.1 to 0.9, with an interval of 0.1. Estimates of the change point and other regression parameters are reported for quantiles (τ = 0.1, 0.3, 0.5, 0.7, and 0.9).

The Structural Equation Model (SEM) can handle multiple dependent variables, analyze the relationship between single variables and scientifically reflect indirect effects between variables (20). SEM can also investigate the direct and indirect effects of LOHS on inpatient costs. The variables affecting inpatient costs in P50 in the quantile regression were used as independent variables, the log inpatient costs as dependent variables, and the log hospitalization days as mediating variables to construct an SEM in IBM SPSS Amos 24.0 software (IBM Corp.). All tests were two-sided, with a test level of 0.05.

## Results

**Figure 1:**
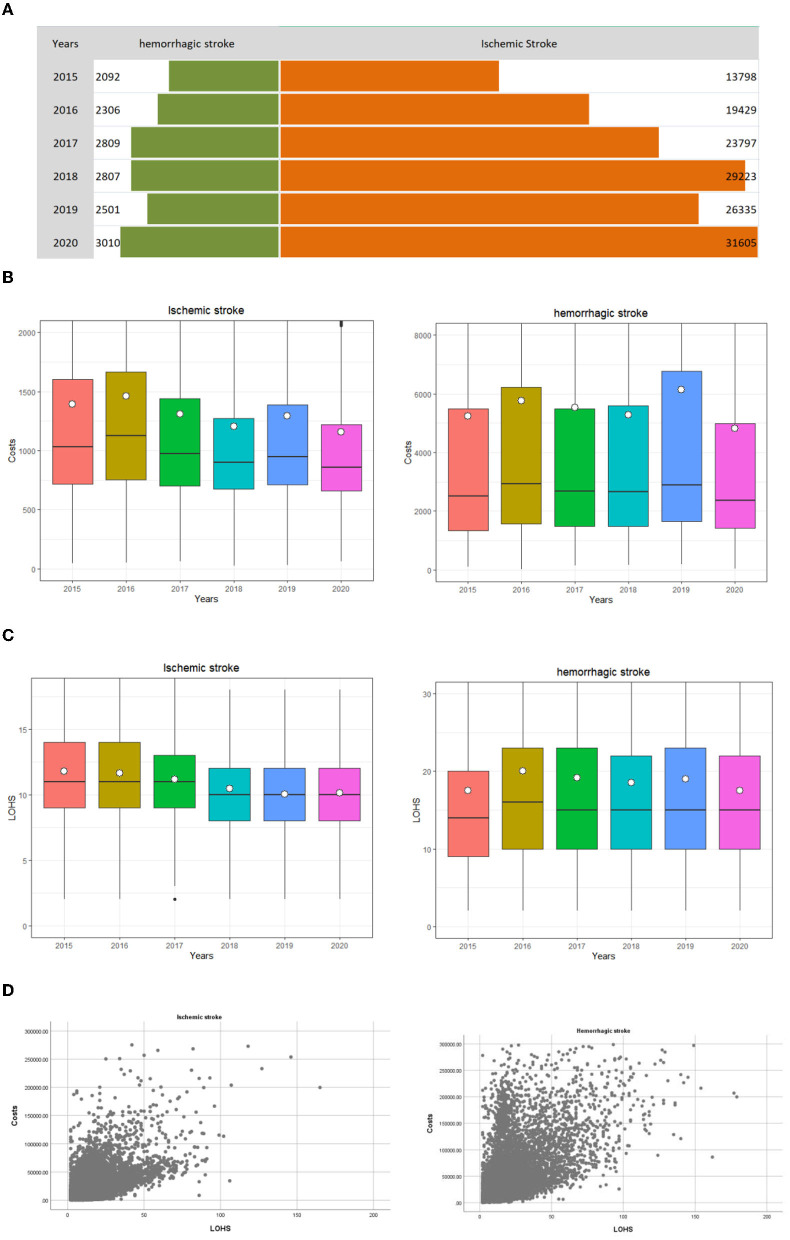
Distribution of inpatients with stroke discharged from 2015 to 2020 in Ningxia, China. The white dots is the per capita expense, and the middle black line of the box plot represents the median, the bottom line of the box plot represents the lower quartile (the first quartile, Q1), indicating that 25% of the overall data is less than the value; the upper border represents the upper quartile (the third quartile, Q3), and 75% of the overall data is less than that value. **(A)** The trend of discharge cases. **(B)** The trend of inpatient costs. **(C)** The trend of length of hospitalization stay. **(D)** The Scatter Chart of hospitalization costs and length of hospitalization stay.

In this study, we analyzed 144,969 stroke samples in Yinchuan between 2015 and 2020, including 15,525 hemorrhagic stroke patients (accounting for 10.7% of the total number of cases) and 129,444 ischemic stroke patients (accounting for 89.3% of the total number of cases). The median LOHS was 10 days (interquartile range: 8–13 days) for ischemic stroke and 15 days (interquartile range: 10–22 days) for hemorrhagic stroke. The trends for discharge cases and LOHS are shown in Figures 1A,C. When discounted by the CPI, the median M (P25, P75) of inpatient costs was $1020 (742–1545) for ischemic stroke and 2813 (1576–6191) for hemorrhagic stroke. The median M (P_25_, P_75_) of inpatient costs for ischemic stroke and hemorrhagic stroke was highest in 2016 at $1,294 (865–1,912) and $3,366 (1,801–7,141), respectively. These values were lowest in 2020 at $936 (699–1,408) and $2,365 (1,429–4,989) respectively (Table 1, Figure 1B). Pearson's correlation analysis showed that the correlation coefficients between the inpatient costs and LOHS for ischemic stroke and hemorrhagic stroke were 0.561 and 0.583, respectively (*p* < 0.001), the Scatter Chart was showed in Figure 1D.

**Table 1:** Discounting of hospitalization expenses for patients with stroke in 2015–2020.

**Discharged years**	**CPI (%)**	**Discount formula**	**Hemorrhagic stroke [P**_**50**_ **(P**_**25**_**–P**_**75**_**),$]**		**Ischemic stroke [P**_**50**_ **(P**_**25**_**–P**_**75**_**),$]**	
			**Before the discount**	**After the discount**	**Before the discount**	**After the discount**
2015 (Y1)	100	103.8%*106.0%*102.4%*101.8%*Y1	2,516 (1,334–5,500)	2,885 (1,530–6,309 )	1,030 (716–1,601)	1,181 (822–1,836)
2016 (Y2)	103.8	103.8%*106.0%*102.4%*101.8%*Y2	2,934 (1,570–6,226)	3,366 (1,801−7,141 )	1,128 (754−1,667)	1,294 (865–1,912)
2017 (Y3)	106.0	106.0%*102.4%*101.8%*Y3	2,671 (1,483-5,502)	2,952 (1,638–6,079)	973 (700–1,439 )	1,076 (773–1,590)
2018 (Y4)	104.3	102.4%*101.8%*Y4	2,652 (1,487–5,599)	2,764 (1,550–5,836)	901 (674–1,273 )	940 (703–1,327)
2019 (Y5)	102.4	101.8%*Y5	2,893 (1,662–6,767)	2,945 (1,692–6,888)	950 (711–1,387)	967 (723–1,412 )
2020 (Y6)	101.8	Y6	2,365 (1,429–4,989)	2,365 (1,429–4,989)	858 (660–1,219)	936 (699–1,408)

Univariate analysis showed that there were no statistical differences in median hospitalization cost of hemorrhagic stroke by gender (*p* = 0.280) and CCI (*p* = 0.393). The median hospitalization cost of hemorrhagic stroke patients differed statistically according age group, hospital level, LOHS, payment type, with or without surgery, patterns of admission, and the method of discharge (*p* < 0.001). All of these influencing factors showed significant differences in terms of the median hospital costs of patients with ischemic stroke (*p* < 0.001) (Table 2). With respect to quantile regression analysis, when we considered patients with ischemic stroke, it was evident that age group had no effect (β = −0.001~0.004, *p* > 0.05) at different quantile points and the payment method was below P10 (β = 0.001, *p* > 0.05) with regards to inpatient costs. However, LOHS (β = 0.764–0.821, *p* < 0.01), with or without surgery (β = 0.038–0.340, *p* < 0.01), CCI (β = 0.001–0.007, *p* < 0.01 or *p* < 0.05), and hospital level (β = 0.154–0.236, *p* < 0.01) had significant positive effects on inpatient costs. Years of discharge (β = −0.003 to −0.016, *p* < 0.01) and patterns of admission (β = −0.014 to −0.105, *p* < 0.01) had significant negative effects on inpatient costs. Patterns of discharge had negative effects on inpatient costs when below P30 (β = −0.005 to −0.021, *p* < 0.01) and had a positive impact on inpatient costs when above P50 (β = 0.017–0.060, *p* < 0.01). For patients with hemorrhagic stroke, hospital level (β = 0.198–0.216, *p* < 0.01), patterns of discharge (β = 0.044–0.054, P < 0.01), payment method (β = 0.03–0.014, *p* < 0.01), LOHS (β = 0.713–0.843, *p* < 0.01), with or without surgery (β = 0.340–0.0560, *p* < 0.01) and CCI (β = 0.003–0.009, *p* < 0.01) had a significant positive impact on inpatient costs. Patterns of admission had a significant negative effect on inpatient costs (β = −0.086 to −0.098, *p* < 0.01). Age group had positive effects on inpatient costs when below P30 (β = 0.000–0.001, *p* < 0.01 or *p* < 0.05) and had a negative impact on inpatient costs when above P90 (β = −0.001, *p* < 0.01), but there was no statistical difference in P50 (β = 0.001, *p* > 0.05) and P70 (β = −0.001, *p* > 0.05) with regards to inpatient costs (Table 3).

**Table 2:** Association between the factors and the hospitalization costs of inpatients with the in the quantile regression model.

**Ischemic stroke**					
**Variables**	**Quantile 0.10**	**Quantile 0.30**	**Quantile 0.50**	**Quantile 0.70**	**Quantile 0.90**
Constant	2.829** (347.886)	2.993** (413.853)	3.102** (404.637)	3.177** (345.967)	3.268** (189.872)
Age	0.000 (0.174)	0.002 (1.310)	0.000 (0.113)	−0.000 (-−0.061)	0.004 (1.388)
Hospital level	0.174** (75.045)	0.154** (78.690)	0.181** (96.068)	0.212** (106.039)	0.236** (75.064)
Year of discharge	−0.009** (–16.118)	−0.016** (–28.625)	−0.016** (–29.127)	−0.013** (–21.750)	−0.003** (–3.153)
Patterns of discharge	−0.021** (–15.774)	−0.005** (–4.224)	0.017** (14.908)	0.032** (25.217)	0.060** (30.069)
Patterns of admission	−0.014** (–6.344)	−0.055** (–31.371)	−0.080** (–44.752)	−0.093** (–45.429)	−0.105** (–29.777)
Payment method	0.001 (1.420)	0.008** (8.933)	0.012** (13.628)	0.016** (16.769)	0.020** (12.596)
Length of stay	0.793** (150.880)	0.821** (176.947)	0.802** (157.601)	0.792** (121.907)	0.764** (58.320)
With or without Surgury	0.038** (8.349)	0.060** (14.613)	0.099** (24.148)	0.166** (36.877)	0.340** (46.978)
CCI	0.010** (16.391)	0.007** (13.392)	0.005** (10.271)	0.004** (6.720)	0.002* (2.373)
*R* ^2^	0.290	0.297	0.318	0.347	0.370
**Hemorrhagic stroke**					
**Variables**	**Quantile 0.10**	**Quantile 0.30**	**Quantile 0.50**	**Quantile 0.70**	**Quantile 0.90**
Constant	2.829** (121.757)	3.013** (166.940)	3.171** (173.416)	3.354** (150.241)	3.627** (99.743)
Age	0.001* (2.575)	0.000** (2.687)	0.000 (0.726)	−0.000 (−0.715)	−0.001** (–2.961)
Hospital level	0.203** (27.544)	0.198** (35.702)	0.207** (37.359)	0.216** (32.626)	0.211** (20.811)
Year of discharge	−0.001 (−0.376)	−0.003* (–2.502)	−0.007** (–4.919)	−0.011** (–6.904)	−0.011** (–4.575)
Patterns of discharge	0.044** (17.085)	0.050** (26.191)	0.052** (28.084)	0.054** (24.739)	0.054** (15.433)
Patterns of admission	−0.098** (–18.493)	−0.091** (–22.946)	−0.086** (–21.212)	−0.089** (–17.234)	−0.094** (–10.781)
Payment method	0.003 (1.218)	0.007** (3.656)	0.008** (3.937)	0.007** (3.132)	0.014** (4.195)
Length of stay	0.843** (110.108)	0.814** (123.625)	0.779** (108.650)	0.743** (80.892)	0.713** (45.031)
With or without Surgury	0.340** (45.115)	0.398** (71.018)	0.442** (79.516)	0.483** (72.239)	0.560** (51.652)
CCI	0.009** (4.152)	0.006** (3.277)	0.008** (4.685)	0.006** (3.293)	0.003 (1.137)
*R* ^2^	0.421	0.449	0.47	0.485	0.462

*p < 0.05 **p < 0.01, The value is “Regression coefficients” in table and “t” in brackets.

**Table 3:** Association between the factors and the length of hospitalization stay with the in the quantile regression model.

**Ischemic stroke**					
**Variables**	**Quantile 0.10**	**Quantile 0.30**	**Quantile 0.50**	**Quantile 0.70**	**Quantile 0.90**
Constant	0.889** (110.029)	0.997** (192.245)	1.077** (239.126)	1.171** (231.089)	1.263** (217.032)
Age	0.001 (0.222)	0.001 (0.634)	−0.000 (–0.000)	−0.005** (–2.703)	−0.007** (–3.572)
Hospital level	0.001 (0.212)	0.014** (7.986)	0.018** (11.830)	0.014** (8.111)	0.015** (7.531)
Year of discharge	−0.010** (–11.267)	−0.013** (–24.559)	−0.015** (–33.427)	−0.017** (–32.553)	−0.016** (–26.247)
Patterns of discharge	−0.075** (–42.577)	−0.028** (–24.745)	−0.020** (–20.889)	−0.013** (–12.213)	0.004** (3.147)
Patterns of admission	0.029** (9.976)	0.009** (5.259)	0.000 (0.001)	−0.011** (–6.444)	−0.023** (–11.850)
Payment method	−0.019** (–14.248)	−0.011** (–13.020)	−0.005** (–7.377)	−0.003** (–3.954)	0.002 (1.734)
With or without Surgury	0.048** (7.702)	0.047** (12.133)	0.051** (15.029)	0.073** (18.840)	0.129** (29.158)
CCI	0.010** (12.257)	0.007** (13.982)	0.006** (14.448)	0.005** (10.070)	0.004** (6.753)
*R* ^2^	0.040	0.027	0.020	0.024	0.036
**Hemorrhagic stroke**					
**Variables**	**Quantile 0.10**	**Quantile 0.30**	**Quantile 0.50**	**Quantile 0.70**	**Quantile 0.90**
Constant	1.001** (26.601)	1.170** (59.534)	1.275** (74.548)	1.398** (70.335)	1.588** (61.691)
Age	−0.002** (–4.026)	−0.001* (–2.376)	−0.001** (–4.023)	−0.002** (–7.533)	−0.002** (–7.391)
Hospital level	0.118** (9.377)	0.052** (7.753)	0.036** (6.147)	0.057** (8.160)	0.115** (12.077)
Year of discharge	0.004 (1.364)	−0.002 (−0.982)	−0.001 (−0.731)	−0.002 (–1.246)	−0.017** (–7.623)
Patterns of discharge	−0.149** (–42.892)	−0.122** (–59.090)	−0.085** (–44.926)	−0.053** (–23.351)	−0.032** (–10.404)
Patterns of admission	0.025** (2.739)	0.019** (3.961)	0.008 (1.860)	0.013* (2.519)	0.021** (2.887)
Payment method	−0.029** (–6.759)	−0.010** (–4.440)	−0.004* (–2.146)	−0.006** (–2.600)	−0.009* (–2.550)
With or without Surgury	0.121** (10.105)	0.135** (20.499)	0.145** (24.610)	0.163** (23.415)	0.224** (23.978)
CCI	0.016** (4.165)	0.007** (3.384)	0.005** (2.672)	0.008** (4.044)	0.020** (7.140)
*R* ^2^	0.153	0.099	0.063	0.054	0.088

*p < 0.05 **p < 0.01, The value is “Regression coefficients” in table and “t” in brackets.

Of the factors influencing LOHS, when considering patients with ischemic stroke, hospital level (β = 0.001–0.018, *p* < 0.01), with or without surgery (β = 0.047–0.129, *p* < 0.01), and CCI (β = 0.004–0.010, *p* < 0.01) had significant positive effects on LOHS. Year of discharge had negative effects on LOHS (β = −0.0017 to −0.010, *p* < 0.01). Age group had no effect when below P50 (β = −0.000–0.001, *p* > 0.05) and had a negative impact on LOHS when above P70 (β = −0.005–0.007, *p* < 0.01). Patterns of discharge had negative effects on LOHS when below P90 (β = −0.013 to −0.075, *p* < 0.01) and had a positive impact when at P90 (β = 0.004, *p* < 0.01). Patterns of admission had positive effects on LOHS when below P30 (β = 0.009~0.029, *p* < 0.01) and had a positive impact when above P70 (β = 0.011~0.023, *p* < 0.01). For patients with hemorrhagic stroke, hospital level (β = 0.036–0.118, *p* < 0.01), patterns of admission (β = 0.008–0.025, *p* < 0.01), with or without surgery (β = 0.121–0.224, *p* < 0.01) and CCI (β = 0.005–0.020, *p* < 0.01) had a significant positive impact on LOHS. Age group (β = −0.001 to −0.002, *p* < 0.01 or *p* < 0.05), patterns of discharge (β = −0.032 to −0.149, *p* < 0.01), payment method (β = −0.004–0.029, *p* < 0.01) had a negative impact on LOHS.

**Figure 2:**
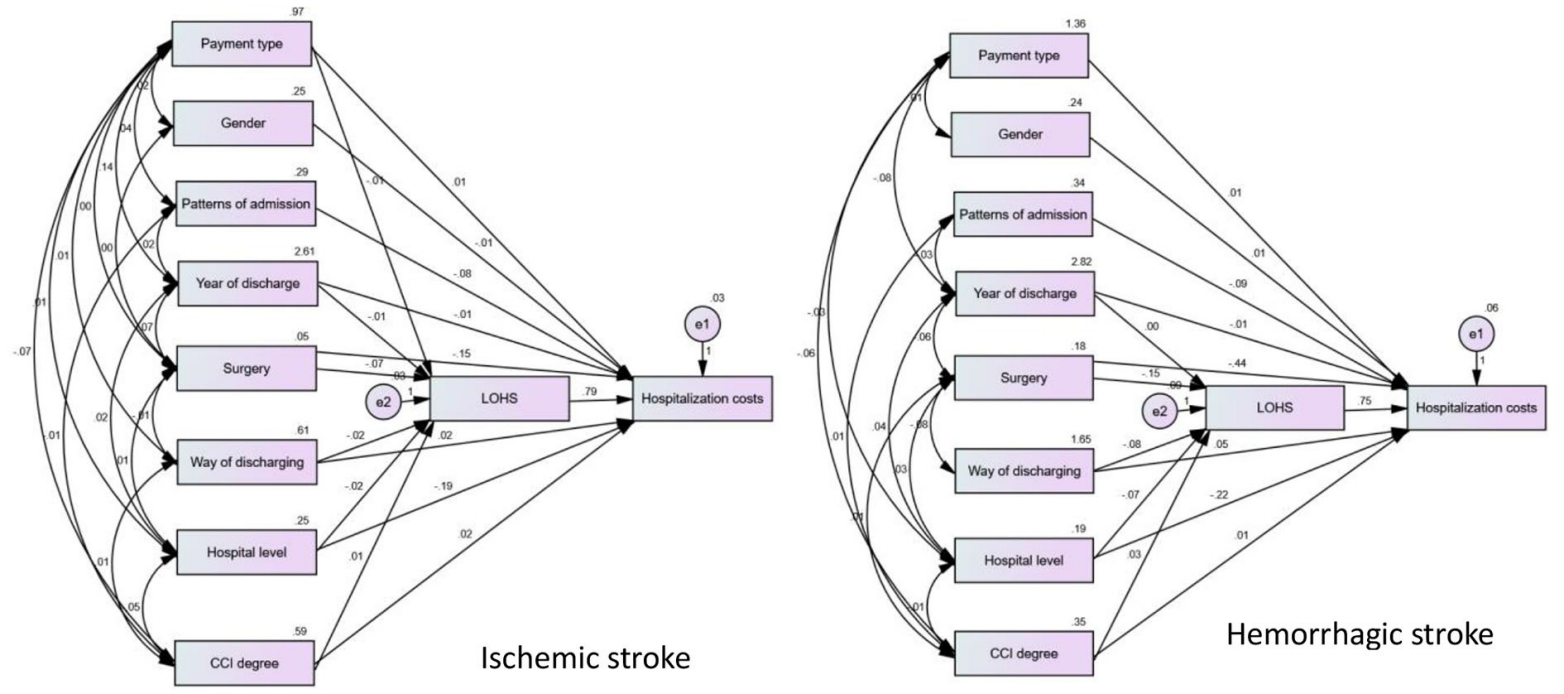
Structural equation model of influencing factors of hospitalization costs in patients with ischemic and hemorrhagic stroke.

According to our structural equation modeling results (Figure 2), the total effect value of LOHS on inpatient costs in patients with ischemic stroke was 0.795 (*p* < 0.001). Of all variables, gender (X2) and patterns of admission (X3) only had direct effects on LOHS (*p* < 0.001) but did not exert effects on the inpatient costs. Correspondingly, the payment type (X1), year of discharge (X4), surgery (X5), method of discharge from hospital (X6), hospital level (X7), and CCI (X8) exerted an impact on inpatient costs *via* two mechanisms: direct effects and indirect effects exerted by LOHS; the path coefficient is shown in Table 4. From the path coefficient, it is evident that payment type (X1) had a positive correlation (0.011, *p* < 0.001) with inpatient costs but had a negative correlation with the LOHS (−0.008, *p* < 0.001); the gross effect was −0.014. Similar results were evident for the method by which patients were discharged from the hospital (X6). The indirect effect of year of discharge (X4), and CCI (X8), was greater than the direct effect.

**Table 4:** Effect decomposition of factors influencing hospitalization costs in patients with ischemic and Hemorrhagic stroke.

**Variable**	**Direct effect**						**Indirect effect**		**Total effect**
	**Path**	**Path coefficient**	***P***	**Path**	**Path coefficient**	***P***	**Path**	**Path coefficient**	
**Ischemic stroke**									
Payment type(X_1_)	X_1_- Y_2_	0.011	<0.001	X_1_- Y_1_	−0.008	<0.001	X_1_- Y_1_- Y_2_	−0.025	−0.014
Gender(X_2_)	X_2_- Y_2_	−0.014	<0.001	X_2_- Y_1_	–	–	X_2_- Y_1_- Y_2_	–	−0.014
Patterns of admission(X_3_)	X_3_- Y_2_	−0.077	<0.001	X_3_- Y_1_	–	–	X_3_- Y_1_- Y_2_	–	−0.077
Year of discharge(X_4_)	X_4_- Y_2_	−0.013	<0.001	X_4_- Y_1_	−0.015	<0.001	X_4_- Y_1_- Y_2_	−0.071	−0.084
Surgery(X_5_)	X_5_- Y_2_	−0.147	<0.001	X_5_- Y_1_	−0.070	<0.001	X_5_- Y_1_- Y_2_	−0.045	−0.192
Way of discharging from hospital(X_6_)	X_6_- Y_2_	0.020	<0.001	X_6_- Y_1_	−0.024	<0.001	X_6_- Y_1_- Y_2_	−0.057	−0.037
Hospital level(X_7_)	X_7_- Y_2_	−0.193	<0.001	X_7_- Y_1_	−0.017	<0.001	X_7_- Y_1_- Y_2_	−0.025	−0.218
CCI degree(X_8_)	X_8_- Y_2_	0.015	<0.001	X_8_-Y_1_	0.015	<0.001	X_8_- Y_1_- Y_2_	0.034	0.049
LOHS(Y_1_)	Y_1_- Y_2_	0.795	<0.001	–	–	–	–	–	0.795
**Hemorrhagic stroke**									
Payment type(X_1_)	X_1_- Y_2_	0.009	<0.001	X_1_- Y_1_	–	–	X_1_- Y_1_- Y_2_	–	0.009
Gender(X_2_)	X_2_- Y_2_	0.011	0.006	X_2_- Y_1_	–	–	X_2_- Y_1_- Y_2_	–	0.011
Patterns of admission(X_3_)	X_3_- Y_2_	−0.092	<0.001	X_3_- Y_1_	–	–	X_3_- Y_1_- Y_2_	–	−0.092
Year of discharge(X_4_)	X_4_- Y_2_	−0.009	<0.001	X_4_- Y_1_	−0.004	0.014	X_4_- Y_1_- Y_2_	−0.010	−0.019
Surgery(X_5_)	X_5_- Y_2_	−0.443	<0.001	X_5_- Y_1_	−0.151	<0.001	X_5_- Y_1_- Y_2_	−0.108	−0.551
Way of discharging from hospital(X_6_)	X_6_- Y_2_	0.049	<0.001	X_6_- Y_1_	−0.084	<0.001	X_6_- Y_1_- Y_2_	−0.184	−0.135
Hospital level(X_7_)	X_7_- Y_2_	−0.220	<0.001	X_7_- Y_1_	−0.074	<0.001	X_7_- Y_1_- Y_2_	−0.055	−0.275
CCI degree(X_8_)	X_8_- Y_2_	0.008	0.026	X_8_-Y_1_	0.028	<0.001	X_8_- Y_1_- Y_2_	0.028	0.036
LOHS(Y_1_)	Y_1_- Y_2_	0.754	<0.001	–	–	–	–	–	0.754

The total effect value of LOHS on inpatient costs in patients with hemorrhagic stroke was 0.754. Of all variables, payment type (X1), gender (X2), and patterns of admission (X3) only had a direct effect on LOH but did not exert an effect on inpatient costs. Year of discharge (X4), surgery (X5), methods by which patients were discharged from hospital (X6), hospital level (X7), and CCI (X8) exerted effects on both inpatient costs and LOHS. Detailed assignments of X1-X8 are shown in Appendix 2.

## Discussion

Over recent years, Ningxia, as with other parts of China, has implemented a series of medical reform policies to control medical costs and to ensure that appropriate medical services are provided to all residents. The cost incurred by stroke inpatients has been declining since 2016; this is due to a series of medical reform policies implemented in the region, such as payment systems for disease diagnosis-related groups and single diseases (21–23). Medical institutions also regulate the behavior of clinicians through clinical pathways, optimize the medical process, reduce inefficient hospitalization days, and control unreasonable costs (24–27). Our study showed the decreasing trend in inpatient costs and LOHS for ischemic stroke, thus confirming the efficacy of these policies and measures. Furthermore, we found that inpatient costs and LOHS are highly relevant, this suggested that the decreasing trend in LOHS for ischemic stroke might have indirectly led to a reduction in inpatient costs.

In this study, we analyzed the factors influencing the hospitalization cost of stroke patients by quantile regression. The quantile regression model showed that LOHS was the largest contributor to inpatient costs; these findings were similar to those of previous studies. In general, for a particular patient, and assuming that his/her condition is certain, the total amount of treatment he/she receives increases as the number of days in hospital increases. An increase in the number of days in hospital was accompanied by a series of basic costs such as more beds, drugs, and nursing care, as well as greater consumption of health resources, thus resulting in higher inpatient costs. When controlling for other variables, some variables such as hospital level (X7) and CCI (X8) showed consistent trends in their effects on LOHS and inpatient costs, thus suggesting a synergistic effect between LOHS and inpatient costs. The inpatient cost and LOHS in tertiary hospitals are higher than those in secondary hospitals because tertiary hospitals have better medical equipment, services, and higher treatment standards when compared to secondary hospitals. Therefore, the tertiary hospitals also admit patients with more complex and serious conditions, thus resulting in higher inpatient costs and inpatient days for patients attending tertiary hospitals. However, in terms of the coefficient of determination, tertiary hospitals had a greater effect on inpatient costs. However, in terms of the coefficient of determination, tertiary hospitals had a greater impact on inpatient costs and a relatively smaller impact on the number of days in the hospital. This also suggested that further enhancement of the hierarchical diagnosis and treatment system could reduce medical burden; for example, by increasing the grading of patients according to their own priorities and difficulty of treatment with different levels of medical institutions undertaking the treatment of different diseases, and by gradually realizing the medical process from general practice to specialization.

Furthermore, we applied AMOS path analysis to construct an SEM to investigate the direct and indirect effects of total patient hospitalization costs. We showed that LOHS contributed the most to the direct effect of hospitalization costs. The CCI score had a greater direct impact on the number of days in the hospital than on the cost of hospitalization while the CCI score indirectly affected the cost of hospitalization via the number of days in the hospital. Comorbidities are a hallmark of stroke that both increase the incidence of stroke and worsen the outcome (28). Higher CCI scores indicate more co-morbidities and more complex conditions. Previous studies have shown that the financial burden of stroke patients with co-morbidities such as hypertension, heart disease, and diabetes is increasing (29). Therefore, optimizing efficiency to reduce LOHS is the best means of reducing the inpatient costs of stroke patients with co-morbidities. In addition, other factors such as year of discharge (X4), surgery (X5), and method of discharge from the hospital (X6), all had a direct effect on hospitalization costs and also indirectly affect hospitalization costs *via* LOHS.

This study described the impact of patient demographic characteristics, payment methods, comorbidities, disease regression, and other indicators, on costs. In the future, we should add other influencing factors such as nursing hours, whether to transfer departments, and the value of the skilled labor provided by medical staff, and further validate the optimization model to propose more refined and targeted measures to effectively control and reduce the inpatient costs of stroke patients.

This study applied a quantile regression model to analyze the factors that can influence stroke hospitalization cost and an SEM to investigate the interrelationship between these factors. The results of this study revealed the direct and indirect effects of LOHS on hospitalization costs and will help to provide effective operational measures to control and reduce hospitalization costs in the future.

## Data availability statement

The raw data supporting the conclusions of this article will be made available by the authors, without undue reservation.

## Author contributions

PL had full access to all of the data in the study and takes responsibility for the integrity of the data and the accuracy of the data analysis and responsible for concept and design. MS and DP drafted the manuscript. PL critically revised the manuscript for important intellectual content. MS carried out the statistical analysis and DP supervised the research. All authors were involved in data acquisition, cleaning, analysis, contributed to the article, and approved the submitted version.

## Funding

This study was funded by the Ningxia Natural Science Foundation of China (Reference: 2020AAC03354) and the Ningxia Hui Autonomous Region Key Research and Development Plan Project (Reference: 2021BEG03099).

## Conflict of interest

The authors declare that the research was conducted in the absence of any commercial or financial relationships that could be construed as a potential conflict of interest.

## Publisher's note

All claims expressed in this article are solely those of the authors and do not necessarily represent those of their affiliated organizations, or those of the publisher, the editors and the reviewers. Any product that may be evaluated in this article, or claim that may be made by its manufacturer, is not guaranteed or endorsed by the publisher.
