# Supplementary material for: The direct and indirect effects of length of hospital stay on the costs of inpatients with stroke in Ningxia, China, between 2015 and 2020: A retrospective study using quantile regression and structural equation models
Source: Front Public Health. 2022 Aug 12;10:881273. doi: 10.3389/fpubh.2022.881273 (PMC9415100; doi:10.3389/fpubh.2022.881273)
Supplement: Supplementary file 1 [file Table_1.docx]

| **Complications (or associated complications)** | **Weight (Score)** |
| --- | --- |
| Myocardial infarction | 1 |
| Congestive heart failure | 1 |
| Peripheral vascular disease | 1 |
| Cerebrovascular disease | 1 |
| Dementia | 1 |
| Chronic pulmonary disease | 1 |
| Connective tissue disease | 1 |
| Ulcer | 1 |
| Mild liver disease | 1 |
| Diabetes | 2 |
| Diabetes with end-organ damage Hemiplegia | 2 |
| Moderate or severe renal disease | 2 |
| Leukemia, lymphoma, any tumor | 2 |
| Moderate or severe liver disease | 3 |
| Metastatic solid tumor | 6 |
| Acquired immune deficiency syndrome | 6 |

**Appendix 1**
