# Supplementary material for: The direct and indirect effects of length of hospital stay on the costs of inpatients with stroke in Ningxia, China, between 2015 and 2020: A retrospective study using quantile regression and structural equation models
Source: Front Public Health. 2022 Aug 12;10:881273. doi: 10.3389/fpubh.2022.881273 (PMC9415100; doi:10.3389/fpubh.2022.881273)
Supplement: Supplementary file 2 [file Table_2.docx]

| Appix 2 Quantitative method and evaluation of factors influencing hospitalization costs | | |
| --- | --- | --- |
| Symbol | Variable | The assignment |
| Y_1_ | LOHS | The log of a specific value |
| Y_2_ | Hospitalization costs | The log of a specific value |
| X_1_ | Payment method | 1=UEBMI,2=URBMI,3=NRCMI,4=Self-paying,5=Others |
| X_2_ | Gender | 1=Male,2=Female |
| X_3_ | Patterns of admission | 1=Out-patient,2=Emergency,3=Transfer,4=Others |
| X_4_ | Year of discharge | 1=2015,2=2016,3=2017,4=2018,5=2019,6=2020 |
| X_5_ | Surgery | 1=Yes,2=No |
| X_6_ | Patterns of discharge | 1=Discharge with doctor's advice,2=Transfer with doctor's advice,3=Transfer to community health service institutes/county medical community,4=Discharge without doctor's advice,5=Death,6=Others |
| X_7_ | Hospital level | 1=Level 3 hospital,2=Level 2 hospital |
| X_8_ | CCI degree | 1=1,2=2,3=3 |
